# The association between pre-hospital antibiotic therapy and subsequent in-hospital mortality in adults presenting with community-acquired pneumonia: an observational study

**DOI:** 10.1186/s41479-018-0047-4

**Published:** 2018-03-25

**Authors:** Biswajit Chakrabarti, Dan Wootton, Steven Lane, Elizabeth Kanwar, Joseph Somers, Jacyln Proctor, Nancy Prospero, Mark Woodhead

**Affiliations:** 1 Aintree University Hospital NHS Foundation Trust, Liverpool, UK; 2 Institute of Infection and Global Health, University of Liverpool, Liverpool, UK; 3 Department of Respiratory Research, Aintree University Hospital NHS Foundation Trust, Liverpool, UK; 4 Institute of Translational Medicine, University of Liverpool, Liverpool, UK; 5 Advancing Quality Alliance, Manchester, UK; 6 Warrington and Halton Hospitals NHS Foundation Trust, Warrington, UK; 7 Department of Respiratory Medicine, Central Manchester University Hospitals NHS Foundation Trust, Manchester, UK; 8 Manchester Academic Health Science Centre and Faculty of Medical and Human Sciences, University of Manchester, Manchester, UK

**Keywords:** Pneumonia, Antibiotics, Community, Mortality, Comorbidity, Severity

## Abstract

**Background:**

The majority of patients with community acquired-pneumonia (CAP) are treated in primary care and the mortality in this group is very low. However, a small but significant proportion of patients who begin treatment in the community subsequently require admission due to symptomatic deterioration. This study compared patients who received community antibiotics prior to admission to those who had not, and looked for associations with clinical outcomes.

**Methods:**

This study analysed the Advancing Quality (AQ) Pneumonia database of patients admitted with CAP to 9 acute hospitals in the northwest of England over a 12-month period.

**Results:**

There were 6348 subjects (mean age 72 [SD 16] years; gender ratio 1:1) admitted with CAP, of whom 17% had been pre-treated with antibiotics. The in-hospital mortality was 18.6% for the pre-treatment group compared to 13.2% in the “antibiotic naïve” group (*p* < 0.001). On multivariate analysis, age, male gender and antibiotic pre-treatment were predictors of in-hospital mortality along with a history of cerebrovascular accident, congestive cardiac failure, dementia, renal disease and cancer. After adjustment for CURB-65 score, age, co-morbidities and pre-treatment with antibiotics remained as independent risk factors for in-hospital mortality (OR 1.43, 95% CI 1.19–1.71).

**Conclusion:**

CAP patients admitted to hospital were more likely to die during admission if they had received antibiotics for the same illness pre-admission. Future studies should endeavor to determine the mechanisms underlying this association, such as microbiological factors and the role of comorbidities. Patients hospitalized with CAP despite prior antibiotic treatment in the community require close monitoring.

**Electronic supplementary material:**

The online version of this article (10.1186/s41479-018-0047-4) contains supplementary material, which is available to authorized users.

## Background

Community-acquired pneumonia (CAP) constitutes a considerable worldwide burden in terms of morbidity, mortality and emergency medical admissions [1]. At the time of presentation to hospital up to 20% of patients diagnosed with CAP have been pre-treated with antibiotics in primary care for symptoms of a lower respiratory tract infection (LRTI) [2, 3]. A paucity of high quality data exists as to whether prior antibiotic therapy in the community is associated with an adverse outcome following hospitalization. However, prior antibiotic therapy is usually a specific criterion excluding entry into antibiotic clinical trials in CAP. CAP is observed with increasing prevalence in the elderly, the immunosuppressed and in those with significant co-morbidity, which are risk factors in themselves for mortality in pneumonia [2, 4]. In any study examining this issue, adequate separation of such variables from prior antibiotic therapy is essential to truly define the contribution of the latter as a potential risk factor in CAP. Advancing Quality (AQ) is a National Health Service (NHS)-recognized initiative set in the northwest of England, which aims to improve standards of healthcare delivery in alignment with national guidelines and encompasses a variety of conditions, including CAP [5]. This UK-based study involves analysis of the AQ Pneumonia database to determine whether in-hospital mortality in adults hospitalized with CAP is associated with prior antibiotic treatment.

## Methods

The AQ Pneumonia program requires participating hospitals to submit data for all cases admitted with a diagnosis of CAP on a rolling monthly basis. The processes underpinning case identification and data entry for the AQ Pneumonia program are outlined in the supplement. For submission to the AQ program, it is necessary for the diagnosis of CAP to be made by a consultant physician following formal medical review within 24 h of hospital admission, along with compatible chest radiograph findings. For each case, key measures integral to the optimal management of CAP are captured; e.g. whether assessment of oxygenation status and CAP severity index were documented, the time to chest radiograph being performed and time to “1st dose inpatient antibiotic administration”, and whether antibiotic selection was appropriate according to local guidelines. The AQ Pneumonia database specifically excludes subjects who do not have an abnormal chest radiograph reported on admission, those referred to palliative care following senior medical review or those on a “palliative care code” on hospital admission, those entered into a clinical trial, those diagnosed with hospital-acquired pneumonia (HAP), and subjects where there is no documentation of a diagnosis of pneumonia following senior consultant medical review after admission.

For each case being submitted to the AQ Pneumonia program, the official admission chest X-ray report (made by a consultant radiologist) is examined by an independent administrator at the time of data entry into the AQ program to ensure that it is compatible with a diagnosis of CAP prior to acceptance. Each hospital participating in the AQ Pneumonia program complies with regular independent NHS audits in order to ensure accuracy of data entry. For each case of CAP submitted to the AQ Pneumonia database, it is also mandatory to record (from analysis of the medical and prescribing records) whether antibiotics were administered in primary care in the 24 h prior to the admission episode, so called “prior-treatment”.

For the analysis, in-patient mortality was defined as and restricted to patients who were recorded as having died at the end of their hospital stay. Patients who were discharged alive and died later are not included in the calculation. In-patient mortality was defined as patients who have a coded discharge method as defined by the NHS Data Dictionary (http://www.datadictionary.nhs.uk). A 30-day readmission was defined as any emergency or unplanned admission to hospital for the same patient within 30 days of the discharge date of the original index admission, while a 30-day readmission due to pneumonia was defined as any readmission based on whether the primary diagnosis code of the readmission is pneumonia, using the same ICD10 list used to identify the initial cohort. Patient comorbidities were identified using the Charlson Comorbidity Index (CCI) as defined in the Standardized Hospital Mortality Guide [6]. This uses ICD10 diagnosis codes in the patient spell to identify if the patient has any of the 17 different comorbidities listed, in which each comorbidity is allocated a different weight according to the burden of disease. The total comorbidity score is the sum weight of the patient’s comorbidities. For this study, analysis of all the CAP cases submitted by the 9 participating hospitals in the AQ Pneumonia program for a 12-month period (April 2016–2017) was undertaken. The 9 hospitals studied were required to submit all cases of CAP to the AQ Pneumonia program during the study period. The analysis was performed by a group comprising respiratory physicians, a senior business intelligence analyst, a medical statistician, two healthcare managers and a respiratory specialist nurse.

### Statistical analysis

Demographic information was summarized using mean and standard deviations for continuous data, unless the data was not normally distributed. In this case, the median and interquartile range was used to describe the average and variability in the data. Categorical data was presented using counts and percentages. The data was stratified depending on whether the patients had been on antibiotic treatment prior to admittance. Following this, formal hypothesis tests (using Pearson’s chi-squared test) were undertaken to investigate possible differences between comorbidities and home antibiotic prescribing, and then between comorbidities and death. Logistic regression was used to construct a multivariate model, which combined demographic variables and comorbidities, along with antibiotic prescribing, to determine the major predictors of death. A further series of logistic regression models were constructed to determine the risk of death for individual comorbidities and antibiotic prescribing. Participant age and, where available, severity of condition (CURB-65) were also included as significant covariates in these models. To complete the analysis, antibiotic home prescribing and the risk of re-admittance with pneumonia was investigated using the chi-squared test. In all analysis, results were considered significant if *p* < 0.05. All analysis was undertaken using SPSS version 22.

## Results

A total of 6348 adults admitted with CAP were submitted to the AQ Pneumonia database during the study period. The study population demographics are outlined in Table 1. The in-hospital mortality for the entire cohort was 14.4% (916/6348). There were 1059 subjects (17%) “pre-treated” with antibiotics prior to admission and 4973 (78%) who were not “pre-treated”. It was not possible to determine whether antibiotics had been administered pre-admission in 316 (5%) subjects.

**Table 1:** Demographics of the study population

Age (mean/SD)	72 (16) years
Gender	3201 (50.4%) female
	3147 (49.6%) male
Length of stay (median/IQR)	5 (3–11) days
Charlson Comorbidity Index (median/IQR)	6 (3–17)
Admission “CURB 65” score	*N* (%)
CURB 65 score 0	547 (9%)
CURB 65 score 1	906 (14%)
CURB 65 score 2	1269 (20%)
CURB 65 score 3	992 (16%)
CURB 65 score 4	244 (3.8%)
CURB 65 score 5	35 (0.6%)
CURB 65 score not documented	2355 (37%)

The incidence of specific comorbidities in the study population, along with the ICD-10 codes ascribed to each condition, is listed in the Additional file 1: Table S1*.* There were no subjects with a coded diagnosis of human immunodeficiency virus (HIV) or acquired immune deficiency syndrome (AIDS) in the cohort. Table 2 outlines the variables associated with in-hospital mortality in the entire CAP cohort. In-hospital mortality was associated with advanced age, higher CCI, “pre-treatment” with antibiotics in primary care and a higher CURB-65 score. The in-hospital mortality was 18.6% (197/1059) in the antibiotic “pre-treated” subjects, compared to 13.2% (658/4973) in those not “pre-treated” (chi square 26.80, df = 2; *p* < 0.001). In terms of specific co-morbidity, a history of congestive cardiac failure (CCF), cerebrovascular accident (CVA), myocardial infarction (MI), renal disease, dementia, cancer (including metastatic) and paraplegia were all associated with in-hospital mortality.

**Table 2:** Comparison of subjects who died in-hospital against those surviving to discharge

	Discharged from hospital	Died in-hospital	
	*n* = 5432	*n* = 916	
Charlson Co-morbidity Index	4 (1–14)	14 (4–23)	*P* < 0.001
Gender			
Female	2761 (50.8%)	440 (48%)	*P* = 0.12
Male	2671 (49.2%)	476 (52%)	
Age (mean/SD years)	71 (16)	80 (11)	*P* < 0.001
History of MI *n* (%)	499 (9.2%)	137 (15.0%)	*P* < 0.001
History of CVA *n* (%)	248 (4.6%)	81 (8.8%)	*P* < 0.001
History of Congestive Cardiac Failure *n* (%)	733 (13.5%)	290 (31.7%)	*P* < 0.001
History of Dementia *n* (%)	581 (10.7%)	178 (19.4%)	*P* = < 0.001
History of Diabetes n(%)	1004 (18.5%)	161 (17.6%)	*P* = 0.51
History of Liver Disease *n* (%)	45 (0.8%)	9 (1.0%)	*P* = 0.64
History of Pulmonary Disease *n* (%)	2564 (47.2%)	405 (44.2%)	*P* = 0.09
History of Cancer *n* (%)	642 (11.8%)	166 (18.1%)	*P* < 0.001
History of Diabetes Mellitus with complications *n* (%)	58 (1.1%)	8 (0.9%)	*P* = 0.59
History of Paraplegia *n* (%)	95 (1.7%)	27 (2.9%)	*P* = 0.02
History of Renal Disease *n* (%)	740 (13.6%)	219 (23.1%)	*P* < 0.001
History of Metastatic Cancer *n* (%)	208 (3.8%)	75 (8.2%)	*P* < 0.001
History of Severe Liver Disease *n* (%)	18 (0.3%)	6 (0.7%)	*P* = 0.14
Admission CURB 65 score (*n* = 3993; 2355 cases not documented)	CURB 0: 538 (15.3%)	CURB 0: 9 (1.9%)	*P* < 0.001
	CURB 1: 863 (24.6%)	CURB 1: 43 (8.9%)	
	CURB 2: 1127 (32.1%)	CURB 2: 142 (29.4%)	
	CURB 3: 799 (22.8%)	CURB 3: 193 (40%)	
	CURB 4: 163 (4.6%)	CURB 4: 81 (16.8%)	
	CURB 5: 20 (0.6%)	CURB 5: 15 (3.1%)	

When comparing the antibiotic “pre-treated” cohort to those not “pre-treated” with antibiotics, an elevated CCI score was observed in the “pre-treated cohort” with a non-significant trend observed for female gender. In terms of specific co-morbidity, the incidence of congestive cardiac failure (CCF) and renal disease was significantly higher in the “pre-treated” cohort. No differences were observed in terms of severity of pneumonia (distribution of CURB-65 scores), age, gender or 30 day re-admission rate (see Table 3).

**Table 3:** Comparison of subjects “pre-treated” with antibiotics prior to admission against those not “pre-treated”

	Not pre-treated with antibiotics pre-admission (*n* = 4973)	Pre-treated with antibiotics pre-admission (*n* = 1059)	*P* value
Age	72.41 (16.71)	72.13 (15.88)	0.61
Gender			
Female	2484 (49.9%)	564 (53.3%)	0.051
Male	2498 (50.1%)	495 (46.7%)	
In-hospital Mortality	658 (13)	197 (19)	< 0.001
Length Of Stay (LOS)	5 (2–11)	5 (3–11)	0.15
Admission CURB-65 score (*n* = 3993; 2355 cases not documented)	CURB 0:463 (14%)	CURB 0: 84 (14%)	0.72
	CURB 1: 780 (23%)	CURB 1: 126 (21%)	
	CURB 2: 1073(32%)	CURB 2: 196 (32%)	
	CURB 3: 830 (25%)	CURB 3: 162 (27%)	
	CURB 4: 211(6%)	CURB 4: 33 (5%)	
	CURB 5: 29 (1%)	CURB 5: 6 (1%)	
Charlson Co-morbidity Index	4 (3–16)	7 (3–17)	0.001
History of MI	489 (9.8)	109 (10.3)	0.65
History of CVA	248 (5)	63 (5.9)	0.20
History of CCF	792 (15.1)	197 (18.6)	0.005
History of Pulmonary Disease	2314 (46.5)	498 (47)	0.78
History of Renal Disease	709 (14.3)	190 (17.9)	0.002
History of Diabetes Mellitus	922 (18.5)	183 (17.3)	0.34
History of Diabetes Mellitus with complications	54 (1.1)	11 (1)	0.89
History of Dementia	578 (11.6)	144 (13.6)	0.07
History of Paraplegia	93 (1.9)	25 (2.4)	0.30
History of Liver Disease	49 (1)	4 (0.4)	0.054
History of Severe Liver Disease	20 (0.4)	3 (0.3)	0.57
History of Cancer	633 (12.7)	133 (12.6)	0.88
History of Metastatic Cancer	224 (4.5)	48 (4.5)	0.97

After applying multivariate analysis (Table 4), the following variables emerged as significant predictors of in-hospital mortality: age, male gender, antibiotic pre-treatment, along with the following co-morbidities: CVA, CCF, dementia, renal disease and cancer. When controlling for age, pre-treatment with antibiotics and severity, gender was not a significant variable in these models. The comorbidities MI (adjusted odds ratio [aOR]1.40 [95% CI 1.09–1.79]), CVA (aOR 1.66 [95% CI 1.21–2.17]), CCF (aOR 2.06 [95% CI 1.69–2.51]), dementia (aOR 1.36 [95% CI 1.09–1.70]), renal (aOR 1.35 [95% CI 1.09–1.66]), metastatic cancer (aOR 2.55 [95% CI 1.66–4.07]) and cancer (aOR 1.57 [95% CI 1.21–2.05]) were all significant predictors of death. In each of these models antibiotic prescribing was also a significant predictor of death (aOR 1.43 [95% CI 1.19–1.71]).

**Table 4:** Multivariate analysis of variables associated with in-hospital mortality

Variable	Adjusted odds ratio	95% confidence interval	Significance
Age	1.04	(1.03, 1.04)	*P* < 0.001
Male Gender	1.17	(1.01, 1.37)	*P* = 0.04
Pre-treatment with antibiotics	1.43	(1.19, 1.71)	*P* = 0.001
History of Cerebrovascular Accident (CVA)	1.55	(1.17, 2.05)	*P* = 0.002
History of Congestive Cardiac Failure (CCF)	2.28	(1.91, 2.71)	*P* < 0.001
History of Dementia	1.42	(1.15, 1.74)	*P* = 0.001
History of Renal Disease	1.27	(1.06, 1.54)	*P* = 0.01
History of Cancer	1.73	(1.41–1.72)	*P* < 0.001

When the analysis was restricted to only those patients with a CURB-65 score (3993 subjects), the CURB-65 score became a significant predictor of mortality (Table 5). Although it should be noted that as the CURB-65 variable only contained 6 categories, the variable was included in the model as 5 dummy indicator variables with the CURB-65 “Zero” score (547 observations) taken as reference. For this variable, the aOR ranged from 1.5 to 16.9, although the 95% confidence interval for the CURB-65 score of 5 was wide (6.2–45.8) due to there being only 35 observations in this category. Of the demographic variables age (aOR 1.03 [95% CI 1.02–1.04]), CCI (aOR 1.02 [95% CI 1.00–1.03]) and pre-treatment with antibiotics (aOR 1.54 [95% CI 1.20–1.99]) were significant predictors of mortality along with three co-morbidity indicators: CCF (aOR 1.62, [95% CI 1.22–2.17]), cancer (aOR 1.40 [95% CI 1.01–1.95]) and metastatic cancer (aOR 1.88 [95% CI 1.03–3.47]).

**Table 5:** Analysis of subjects with a documented CURB-65 score

Variable	Regression coefficient	Adjusted odds ratio	95% confidence interval	Significance
Age	0.03	1.03	(1.02, 1.04)	*P* < 0.001
CCI	0.02	1.02	(1.00, 1.03)	*P* = 0.008
Pre-treatment with antibiotics	0.43	1.54	(1.20, 1.99)	*P* = 0.001
History of CCF	0.49	1.62	(1.22, 2.17)	*P* = 0.001
History of Cancer	0.34	1.40	(1.01, 1.95)	*P* = 0.046
History of metastatic cancer	0.64	1.88	(1.03, 3.47)	*P* = 0.04
CURB65 0		Reference		
CURB65 1	0.42	1.53	(0.72, 3.26)	*P* = 0.27
CURB65 2	1.05	2.85	(1.37, 5.92)	*P* = 0.005
CURB65 3	1.56	4.77	(2.38, 9.97)	*P* < 0.001
CURB65 4	2.25	9.48	(4.37, 20.56)	*P* < 0.001
CURB65 5	2.83	16.88	(6.22, 45.77)	*P* < 0.001

Of the 5432 subjects who survived the index admission, 1095 (21%) were readmitted within 30 days. Of these, 362 (33.1%) were specifically coded for pneumonia as a primary diagnosis. The administration of pre-hospital antibiotics was not associated with a significantly increased risk of 30-day readmission either due to any cause or specifically due to pneumonia (Table 6).

**Table 6:** The relationship between 30-day readmission and “pre-treatment” with antibiotics

	Readmission due to any cause		
	No	Yes	
Not “pre-treated”	4118 (82.4%)	878 (82.5%)	*P* = 0.94
“Pre-treated”	855 (17.6%)	181 (17.5%)	
	Readmission with Pneumonia		
	No	Yes	
Not “pre-treated”	3877 (83.4%)	438 (83.1%)	*P* = 0.88
“Pre-treated”	273 (16.6%)	89 (16.9%)	

## Discussion

This study has shown that in adults admitted to hospital with a diagnosis of CAP, the in-hospital mortality was higher in those who had received antibiotic treatment immediately prior to admission. This finding remained even when controlling for variables that have traditionally been shown to be of prognostic value in CAP, such as co-morbidity, age, and the CURB-65 score. The clinical relevance of this lies in the fact that currently, national guidance for the management of CAP does not recommend a change in management strategy when clinicians in the emergency department encounter a patient with pneumonia “pre-treated” with antibiotics in the community. The evidence associating the outcome from CAP specifically with antibiotic pre-treatment has been conflicting to date [7–14]. A single-centre study of 2179 subjects hospitalized with CAP reported that 17% had been treated with antibiotics prior to admission, a proportion similar to the current study’s findings, yet no significant differences were noted in 30-day mortality, length of stay, or incidence of intensive care unit (ICU) admission, although the frequency of bacteremia was less in the “pre-treatment” group [7]. However, in contrast to the current study, the “prior antibiotic” group had less in terms of co-morbidity in the form of diabetes and cardiac disease as well as a significantly lower proportion of “severe” pneumonia according to CURB-65 criteria, which may explain the authors’ findings. In contrast to the current study’s cohort, this group had less severe pneumonia as evidenced by lower CURB65 scores. Their patients also had less diabetic and cardiovascular comorbidity. The issue of prior outpatient therapy was addressed by Van de Garde who reported that of 1096 subjects hospitalized for CAP over a 5 year period in the Netherlands, 27% had been “pre-treated” with antibiotics with no significant difference reported in mortality [8]. However, the diagnosis of CAP was based on ICD coding which has been shown to carry limitations in diagnostic accuracy [9]. In contrast, the current study’s AQ cohort had a more stringent process to ensure an accurate diagnosis of CAP; i.e. confirmed radiological diagnosis of CAP verified by an independent reviewer at the time of data entry, coupled with the requirement that a documented diagnosis of CAP had to have been made by a senior physician within the first 24 h following admission to hospital. Similarly, a smaller retrospective North American study of 733 subjects admitted with CAP to 2 centres did not report a significant difference in mortality in the 17% of subjects who had received antibiotics prior to admission [13]. However, the definition of “prior antibiotic therapy” in this study included subjects who had received antibiotics within 30 days of the admission, as opposed to the current study where only those who were being treated with antibiotics within 24 h of admission were captured. Furthermore, the North American study excluded those who were nursing home residents and 32% of the pre-treated cohort was administered fluoroquinolones prior to admission, which would not be normal practice in the UK where the prescription of antibiotics in the primary care setting is more tightly regulated. A prospective single-centre study of 3364 subjects reported that similar to the findings of this study, 18% of subjects had been administered antibiotics in the 24 h prior to admission [10]. In contrast to the data from the current study, the authors reported improved outcomes in the “pre-treated” cohort, namely a reduced need for mechanical ventilation and a decrease in the incidence of septic shock, but no difference in overall mortality. However, such a difference may be explained due to this cohort having a higher incidence of “low severity” pneumonia (defined as a CURB score of 0–1) standing at 50% and 56% in the non-pretreated and pretreated groups, respectively, in comparison to 14% observed in both groups of the current cohort. Furthermore, as with earlier studies, the range of antibiotics administered in primary care settings was more diverse when compared to the UK (including cefotaxime and quinolones) and in addition, this study specifically excluded nursing home residents. Similarly, in the earlier described study of 733 pneumonia cases admitted to 2 US hospitals, the 30-day mortality in the entire cohort was reported to be 8.1% and 55% of the study population had pneumonia classified as “low risk”, which suggests there may be differences in the study population from that represented in the current study’s cohort [13]. Another retrospective study of more than 18,000 patients aged over 65 years hospitalized with pneumonia reported that 24.4% had received pre-hospital antibiotic therapy [14]. This study reported that pre-hospital antibiotics were likely to have been administered if subjects were female gender and from a “skilled nursing facility” and were more likely to have had decreased pneumonia severity when compared with those not receiving antibiotics pre-hospital, whilst the current study reported a non-significant trend to female gender and pre-hospital antibiotic therapy but not with pneumonia severity when using CURB-65 as opposed to the pneumonia severity index (PSI). Unsurprisingly, the authors reported that the subjects administered pre-hospital antibiotics were less likely to be admitted to intensive care although this may be explained by this group presenting with less severe CAP according to PSI score. Interestingly, the “pre-treated” group had higher 30-day mortality if antibiotics were administered within 8 h of hospital admission compared to administration beyond 8 h from admission. As in previous studies, the in-hospital mortality was considerably lower than reported in the current study’s cohort, standing at 7%, which again suggests that the study population may have been less “sick” at presentation than the cohort from this current study.

There are a number of potential mechanisms to explain these observations and whether these differences in observed outcome can be explained solely by treatment failure at a microbiological level or reflect a complex interplay between co-morbidities and the host microbiome require further study. The subjects admitted following antibiotic treatment in primary care could have represented a “sicker” cohort with a greater burden of co-morbidity, as evidenced by the fact that there was a higher incidence of cardiac failure and renal disease in this group. However, interestingly, the CURB-65 score, a widely used measure to risk stratify CAP at admission in UK hospitals, was not found to differ significantly between the 2 groups nor did age itself when taken separately. Despite there being no observed difference in CAP severity, as measured by CURB65 score, between the pre-treated and non-pre-treated groups it is possible there are unmeasured markers of severity. The study observed a statistically significant increase in the frequency of renal and cardiac disease in pre-treated patients. These conditions cause fluid overload with associated changes in antibiotic absorption via the gut and across “wet” lungs. These conditions cause increased susceptibility to CAP and possible reduced efficacy of therapy and as a consequence primary care physicians are more likely to prescribe prompt antibiotics. Whilst the UK has a low level of pneumococcal resistance to beta-lactam antibiotics, a proportion of this cohort were also diagnosed with underlying chronic lung disease which may suggest the presence of an expanded range of pathogens causing CAP such as *Haemophilus influenzae*, and *Pseudomonas aeruginosa* [15]. An intriguing mechanism to explain these findings may lie in the fact that the traditional concept of a “sterile” lung has been challenged of late, and the pathogenesis of pneumonia in an individual has been linked to alterations in the lung microbiome [16]. Further studies are required to better understand the effect prior antibiotic therapy has in terms of causing imbalances in the lung biome in the specific setting of inpatients hospitalized with CAP. Irrespective of any potential mechanism, the authors propose that clinicians adopt a degree of vigilance when managing this cohort of patients following hospital admission and that such patients warrant close monitoring with early input from senior physicians and clinical microbiology. However, whether antibiotic prescribing should differ from established guidance when managing such a cohort of CAP patients from those who have not received antibiotics prior to admission requires more detailed prospective study [17].

A particular strength of this study is that the Advancing Quality database is a nationally recognized initiative adopted by multiple NHS hospitals in the northwest of England and data quality is subject to strict audit and regulation. The diagnosis of CAP was more robust in the study as it was not based solely on ICD coding as in previous studies, but from analysis of medical records documenting a diagnosis of CAP made by a consultant physician, exclusion of those with a normal chest radiograph from the AQ database, and an independent review at the time of data entry ensuring the official chest X-ray report was compatible with a diagnosis of CAP. Furthermore, subjects with a “Palliative” code were also excluded from the AQ analysis, as were cases of HAP and “Aspiration” pneumonia; hence, for the cases entered, CAP was more likely to play a more significant role in terms of contributing to in-hospital mortality. The fact that this multi-centre study involved 9 hospitals and more than 6000 subjects adds to the strength of this data. Furthermore, all the 9 hospitals studied were required to submit all cases of CAP to the AQ program during the study period, thus reducing the possibility of selection bias. The age, pattern of comorbidities and in-patient mortality was similar to that reported in the BTS audit cohort although lung disease was more frequent in this current group [1].

Limitations to this study include that antibiotic pre-treatment was ascertained by inspection of medical and hospital pharmacy records, which may have missed certain cases where documentation did not take place. The analysis did not take into account differences in duration of antibiotic pre-treatment as a predictor of outcome; i.e. the study did not distinguish between the scenario of a patient admitted with CAP having been administered simply one dose of antibiotic prior to admission (therefore not necessarily deemed to have “failed” an antibiotic course in the community), compared to a different situation where a patient receives several courses of antibiotics in the weeks prior to admission yet still requires admission due to inadequate resolution of symptoms. Furthermore, the dose and type of antibiotic prescribed in primary care was not studied and this could have influenced outcome. However, in the UK, prescribing options in primary care are highly regulated according to national guidelines as opposed to “physician discretion” where the vast majority of patients will have received amoxicillin either as monotherapy or with clarithromycin or monotherapy with doxycycline or clarithromycin [18]. This study’s dataset did not contain any specific information regarding microbiology testing from the cohort during the hospital admission, hence specific associations between microbiology results and the impact of antibiotic pre-treatment on outcomes were not examined. This requires detailed prospective study. A further limitation is that in this study’s cohort, the “CURB-65 score” was not documented in 37% of cases. However, when taking only those cases where the “CURB-65” score had been documented, antibiotic pre-treatment still emerged as significant following multivariate analysis. This limitation should also be balanced with the large sample size of the study population coupled with the fact that the statistical analysis controlled for comorbidity.

Furthermore, whilst, the CURB-65 score itself represents a standard of care in terms of CAP severity assessment, the relationship between antibiotic pre-treatment and other markers of adverse outcome (such as ICU admission, incidence of multi-organ failure and oxygenation) was not examined. In addition, whilst this study did control for key conditions known to result in immunosuppression, such as hematological malignancy, it did not directly study the effect of immunosuppressive and other medications associated with an increased incidence CAP [19, 20]. Another limitation is that this dataset did not record detailed surrogate markers reflecting the severity of co-morbidity which may have influenced outcome, such as nursing home residence, frequency of hospital admissions in the preceding 12 months, or number of outpatient attendances; e.g. for dialysis. However, the 30-day readmission rate due to any cause was not found to differ between the antibiotic pre-treatment group and those subjects not pre-treated with antibiotics prior to admission.

## Conclusion

In summary, this study reports a higher mortality for subjects hospitalized with CAP who had received antibiotic therapy in the community immediately prior to hospital admission, a relationship maintained independent of comorbidity and disease severity. Such patients require close clinical monitoring in hospital with early specialist input. Future prospective studies should endeavor to determine the underlying mechanisms responsible for this observation focusing on factors such as disease etiology, microbiological variables, alterations to the lung microbiome, and adherence to therapy.

## Additional file

Additional file 1: **Table S1.** Incidence of Co-morbidity in the study population. (DOCX 67 kb)

## Abbreviations

AIDS: Acquired Immune Deficiency Syndrome
aOR: Adjusted Odds Ratio
AQ: Advancing Quality
CAP: Community Acquired Pneumonia
CCF: Congestive Cardiac Failure
CCI: Charlson Comorbidity Index
CVA: Cerebrovascular Accident
HAP: Hospital Acquired Pneumonia
HIV: Human Immunodeficiency Virus
ICD: International Classification Of Diseases
ICU: Intensive Care Unit
LOS: Length Of Stay
LRTI: Lower Respiratory Tract Infection
MI: Myocardial Infarction
NHS: National Health Service
PSI: Pneumonia Severity Index
SuS PbR: Secondary Uses Service Payment by Results

## References

[1] Lim WS, Smith DL, Wise MP, Welham SA (2015). British Thoracic Society community acquired pneumonia guideline and the NICE pneumonia guideline: how they fit together. Thorax.

[2] Fine MJ, Auble TE, Yealy DM, Hanusa BH, Weissfeld LA, Singer DE (1997). A prediction rule to identify low-risk patients with community-acquired pneumonia. N Engl J Med.

[3] Minogue MF, Coley CM, Fine MJ, Marrie TJ, Kapoor WN, Singer DE (1998). Patients hospitalized after initial outpatient treatment for community-acquired pneumonia. Ann Emerg Med.

[4] Lim WS (2003). Defining community acquired pneumonia severity on presentation to hospital: an international derivation and validation study. Thorax.

[5] Sutton M, Nikolova S, Boaden R, Lester H, McDonald R, Roland M (2012). Reduced mortality with hospital pay for performance in England. N Engl J Med.

[6] Charlson ME, Pompei P, Ales KL, MacKenzie CR (1987). A new method of classifying prognostic comorbidity in longitudinal studies: development and validation. J Chronic Dis.

[7] Simonetti AF, Viasus D, Garcia-Vidal C, Grillo S, Molero L, Dorca J, Carratala J (2014). Impact of pre-hospital antibiotic use on community-acquired pneumonia. Clin Microbiol Infect.

[8] Van de Garde EMW, Souverein PC, van den Bosch JMM, Deneer VHM, Goettsch WG, Leufkens HGM (2006). Prior outpatient antibacterial therapy as prognostic factor for mortality in hospitalized pneumonia patients. Respir Med.

[9] Van de Garde EMW, Oosterheert JJ, Bonten M, Kaplan RC, Leufkens HGM (2007). International classification of diseases codes showed modest sensitivity for detecting community-acquired pneumonia. J Clin Epidemiol.

[10] Amaro R, Sellares J, Polverino E, Cilloniz C, Ferrer M, Fernandez-Barat L, Mensa J, Niederman M, Torres A (2017). Antibiotic therapy prior to hospital admission is associated with reduced septic shock and need for mechanical ventilation in patients with community-acquired pneumonia. J Inf Secur.

[11] Johnson D, Carriere KC, Jin Y, Marrie T (2004). Appropriate antibiotic utilization in seniors prior to hospitalization for community-acquired pneumonia is associated with decreased in-hospital mortality. J Clin Pharm Ther.

[12] Meehan TP, Fine MJ, Krumholz HM, Scinto JD, Galusha DH, Mockalis JT (1997). Quality of care, process, and outcomes in elderly patients with pneumonia. JAMA.

[13] Mortensen EM, Restrepo MI, Pugh JA, Anzueto A (2008). Impact of prior outpatient antibiotic use on mortality for community acquired pneumonia: a retrospective cohort study. BMC Res Notes.

[14] Houck PM, Bratzler DW, Nsa W, Ma A, Bartlett JG (2004). Timing of antibiotic administration and outcomes for Medicare patients hospitalized with community-acquired pneumonia. Arch Intern Med.

[15] Health Protection Report Vol 10 no 40; 18^th^ November 2016.

[16] Dickson RP, Erb-Downward JR, Huffnagle GB (2013). The role of the bacterial microbiome in lung disease. Expert Rev Respir Med [Internet].

[17] Van de Garde EMW, Natsch S, Prins JM, van der Linden PD (2015). Antibiotic prescribing on admission to patients with pneumonia and prior outpatient antibiotic treatment: a cohort study on clinical outcome. Br Med J.

[18] NHS, Centre for Clinical Practice at NICE. Respiratory tract infections – antibiotic prescribing [Internet]. NICE Clinical Guidelines. 2008; Available from: http://www.ncbi.nlm.nih.gov/pubmed/21698847

[19] Suissa S, Ernst P (2017). Precision medicine urgency: the case of inhaled corticosteroids in COPD. Chest.

[20] Ramsay EN, Pratt NL, Ryan P, Roughead EE (2013). Proton pump inhibitors and the risk of pneumonia: a comparison of cohort and self-controlled case series designs. BMC Med Res Methodol [Internet].

