# Supplementary material for: The association between pre-hospital antibiotic therapy and subsequent in-hospital mortality in adults presenting with community-acquired pneumonia: an observational study
Source: Pneumonia (Nathan). 2018 Mar 25;10:2. doi: 10.1186/s41479-018-0047-4 (PMC5866909; doi:10.1186/s41479-018-0047-4)
Supplement: Supplementary file 1 — Table S1. Incidence of Co-morbidity in the study population. (DOCX 67 kb) [file 41479_2018_47_MOESM1_ESM.docx]

The process of case identification and data capture for the Advancing Quality (AQ) Pneumonia program

The AQ pneumonia program uses the Secondary Uses Service Payment by Results (SuS PbR) data extract from NHS Digital to identify the cohort and this is detailed in the supplementary document. This is done using the International Classification Of Diseases (ICD)10 coding for pneumonia. The SuS PbR data also contains detail about patient demographics (such as age and gender) and information about the hospital admission such as whether the patient was discharged alive or died at the end of the spell. Once the cohort has been identified, participating hospitals are required to review the patients and answer additional qualification questions to ensure that the admission was suitable for the AQ CAP measures. The hospital then submits the additional performance data for the validated patient set.

Table 1 (Supplement): Incidence of Co-morbidity in the study population

| Co-morbidity | Incidence in study cohort  N (%) |
| --- | --- |
| MI (Codes I21, I22, I23, I252, I258) | 636 (10%) |
| CVA (Codes G450, G451, G452, G454, G458, G459, G46, I60-I69) | 329 (5.2%) |
| Congestive Cardiac Failure (Codes I50) | 1023 (16.1%) |
| Pulmonary Disease  (Codes J40-J47, J60-J67) | 2969 (46.8%) |
| Renal Disease (Codes I12, I13, N01, N03, N052-N056, N072-N074, N18, N19, N25) | 959 (15.1%) |
| Diabetes Mellitus (Codes E101, E105, E106, E108, E109, E111, E115, E116, E118, E119, E131, E136, E138, E139, E141, E145, E146, E148, E149) | 1165 (18.4%) |
| Diabetes Mellitus with complications  (Codes E102, E103, E104, E107, E112, E113, E114, E117,E132, E133, E134, E137,E142, E143, E144, E147) | 66 (1%) |
| Dementia  (Codes F00, F01, F02, F03, F051) | 759 (12%) |
| Paraplegia (Codes G041, G81, G820, G821, G822) | 122 (1.9%) |
| Liver Disease (Codes K702, K703, K717, K73, K74) | 54 (0.9%) |
| Severe Liver Disease (Codes K721, K729, K766, K767) | 24 (0.4%) |
| Cancer (C00-C76, C81-C97) | 808 (12.7%) |
| Metastatic Cancer (Codes C77, C78, C79, C80) | 283 (4.5%) |
